# High Incidence of Ischemic Stroke Occurrence in Irradiated Lung Cancer Patients: A Population-Based Surgical Cohort Study

**DOI:** 10.1371/journal.pone.0094377

**Published:** 2014-04-07

**Authors:** Shih-Kai Hung, Moon-Sing Lee, Wen-Yen Chiou, Ching-Chih Lee, Yi-Chun Chen, Chun-Liang Lai, Nai-Chuan Chien, Wen-Lin Hsu, Dai-Wei Liu, Yu-Chieh Su, Szu-Chi Li, Hung-Chih Lai, Shiang-Jiun Tsai, Feng-Chun Hsu, Hon-Yi Lin

**Affiliations:** 1 Departments of Radiation Oncology, Buddhist Dalin Tzu Chi Hospital, Taiwan, ROC; 2 Department of Otolaryngology, Buddhist Dalin Tzu Chi Hospital, Taiwan, ROC; 3 Division of Nephrology, Buddhist Dalin Tzu Chi Hospital, Taiwan, ROC; 4 Division of Chest Medicine, Buddhist Dalin Tzu Chi Hospital, Taiwan, ROC; 5 Division of Thoracic Surgery, Buddhist Dalin Tzu Chi Hospital, Taiwan, ROC; 6 Division of Hematology-Oncology, Buddhist Dalin Tzu Chi Hospital, Taiwan, ROC; 7 Department of Radiation Oncology, Buddhist Tzu Chi General Hospital, Hualien, Taiwan, ROC; 8 School of Medicine, Tzu Chi University, Hualien, Taiwan, ROC; 9 Institute of Molecular Biology, National Chung Cheng University, Min-Hsiung, Chia-Yi, Taiwan, ROC; Univesity of Texas Southwestern Medical Center at Dallas, United States of America

## Abstract

**Background and Purpose:**

A high risk of stroke occurrence has been reported in several types of irradiated cancer patients. However, clinical data are lacking in irradiated lung cancer patients. The present study intended to explore a risk level of ischemic stroke occurrence in irradiated lung cancer patients.

**Methods:**

A nationwide population-based database obtained from the Taiwan National Health Insurance was analyzed. Between 2003 and 2006, we recruited 560 resected lung cancer patients into two study groups: surgery-plus-irradiation (*n* = 112) and surgery-alone (*n* = 448). Patients treated with chemotherapy were excluded. Propensity score match was used for pairing cases with a ratio of 1∶4. Two-year ischemic-stroke-free survival was defined as the primary endpoint.

**Results:**

Three observations supported a high risk of ischemic stroke occurrence in patients with postoperative irradiation when compared with those patients with surgery alone: first, a high incidence per 1,000 person-year (22.3 versus 11.2, 1.99 folds); second, a low two-year ischemic-stroke-free survival rate (92.2% versus 98.1%, *P* = 0.019); and third, a high adjusted hazard ratio (HR, 4.19; 95% CI, 1.44–12.22; *P* = 0.009). More notably, the highest risk of ischemic stroke occurrence was found in irradiated patients who had diabetes mellitus (HR, 34.74; 95% CI, 6.35->100; *P*<0.0001).

**Conclusions:**

A high incidence of ischemic stroke was observed in irradiated lung cancer patients, especially in those with diabetes mellitus. For these patients, close clinical surveillance and strict diabetes control should be considered. Further studies to define detail biological mechanisms are encouraged.

## Introduction

### High risks of stroke occurrence have been observed in irradiated cancer patients

Stroke is a major disease burden worldwide – once occurred, it may result in significant morbidities and mortality [1]. In the literature, a high risk of stroke occurrence has been reported in several types of irradiated cancer patients, such as head and neck cancers [2], breast cancer [3], and Hodgkin's lymphoma [4]. In these irradiated patients, one treatment point is similar. That is, radiotherapy (RT) is given to cover primary and lymphatic-drainage areas, which include the neck and/or mediastinum [5], [6]. In such a condition, ionizing radiation cannot be avoided to deliver on brain-supplied blood vessels [6], [7]. As a result, late vascular damage and subsequent embolic events may occur [8].

### Little is known about risk of stroke occurrence in irradiated lung cancer patients

Clinically, the mediastinum was irradiated in both lung cancer and Hodgkin's lymphoma patients. However, when compared with irradiated Hodgkin's lymphoma patients [4], [9], [10], reported evidence of stroke risk is largely lacking in irradiated lung cancer patients. From the literature, it is clear that RT-associated stroke is particularly noted in long-term survivors [11], [12]. Thus, this data discrepancy may be partly due to a significant difference of patient survival in these two disease entities. In lung cancer patients, an average shorter survival may obscure an observation of stroke occurrence.

Recently, a high stroke risk has been reported in lung cancer patients. More notably, this risk effect can be early observed at a follow-up time of two years [13]. This finding sheds us a light to explore a potential risk of ischemic stroke occurrence in irradiated lung cancer patients.

### Purpose of the present study

The present study intended to explore whether a risk of ischemic stroke occurrence is really high in irradiated lung cancer patients. Early stage lung cancer patients treated with surgery – but without a component of chemotherapy – were recruited. Of these, patients treated with postoperative radiotherapy were defined as the group of interesting. On the other hand, patients treated with surgery alone were match-paired as intra-cohort controls. Multivariate analysis confirmed a high risk association of ischemic stroke occurrence in irradiated lung cancer patients, especially in those patients with diabetes mellitus.

## Methods

### Database and ethic statement

The present study used a research database obtained from the Taiwan National Health Insurance (TNHI). This database covered medical care of more than 97% of Taiwanese [14], [15]. The human experiments followed here were in accordance with the Helsinki Declaration and were approved by the Institution Review Board (IRB) of our institution, i.e. Buddhist Dalin Tzu Chi Hospital (approved number, B10001019). The IRB waived requirement for written informed consents from patients involved because researchers cannot contact with individual patients from this de-identified database [13], [16].

### Patient allocation and study groups

**Figure 1:**
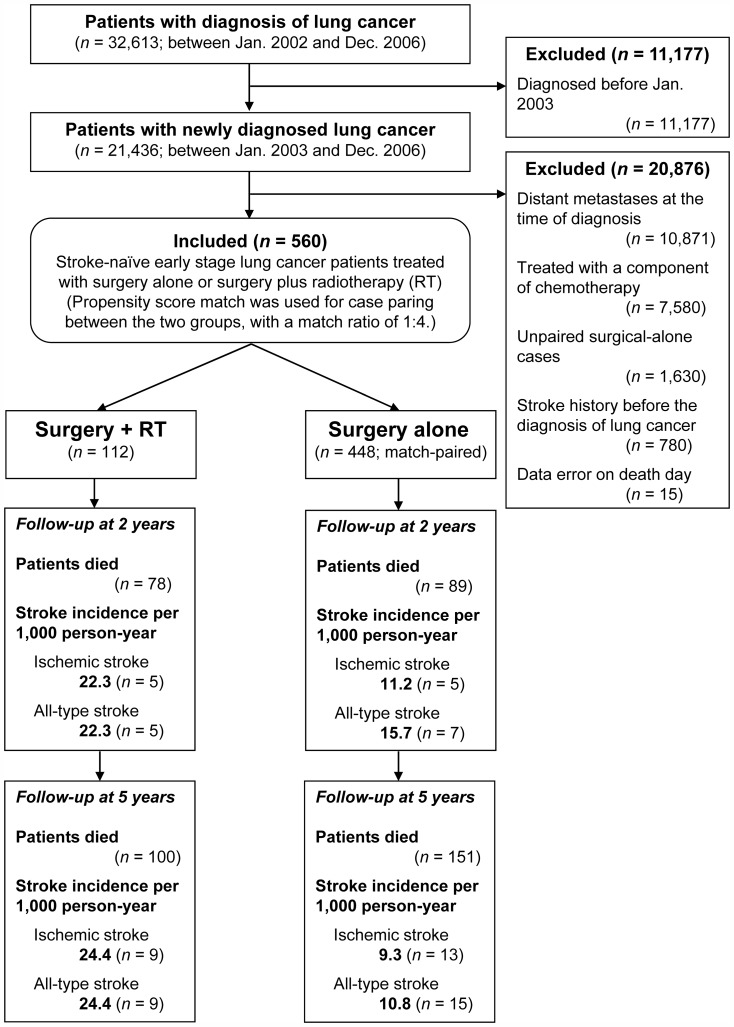
Flow chart of patient allocation. With respect to patients treated with surgery plus radiotherapy (RT), patients treated with surgery alone were match-paired by using a ratio of 1∶4. **Note**: Data coded errors were validated by using the data subset of Registry for Catastrophe Illness.

Between Jan. 2003 and Dec. 2006, we recruited 560 newly diagnosed early stage lung cancer patients into the following two groups: surgery plus radiotherapy (*n* = 112) and surgery alone (*n* = 448; Fig. 1 and table 1).

**Table 1:** Patient and demographic characteristics according to treatment groups.

	Treatment group, *n* (%)			Total, *n* (%)
	Surgery + RT	Surgery alone	*P* value	
Age			*0.999*	
≤65 years	77 (68.8)	308 (68.8)		385 (68.8)
>65 years	35 (31.5)	140 (31.3)		175 (31.3)
Gender			*0.766*	
Male	84 (75.0)	342 (76.3)		426 (76.1)
Female	28 (25.0)	106 (23.7)		134 (23.9)
Hypertension			*0.464*	
Yes	31 (27.7)	109 (24.3)		140 (25.0)
No	81 (72.3)	339 (75.7)		420 (75.0)
Diabetes mellitus			*0.819*	
Yes	19 (17.0)	72 (16.1)		91 (16.3)
No	93 (83.0)	376 (83.9)		469 (83.8)
CAD*			*0.341*	
Yes	11 (9.8)	32 (7.1)		43 (7.7)
No	101 (90.2)	416 (92.9)		517 (92.3)
Atrial fibrillation			*0.885*	
Yes	1 (0.9)	3 (0.7)		3 (0.5)
No	111 (99.1)	445 (99.3)		557 (99.5)
Geographic region			*0.926*	
Northern	44 (39.3)	187 (41.7)		231 (41.3)
Central	27 (24.1)	111 (24.8)		138 (24.6)
Southern	39 (34.8)	144 (32.1)		183 (32.7)
Eastern	2 (1.8)	6 (1.3)		8 (1.4)
Urbanization level			*0.776*	
Urban	26 (23.2)	109 (24.3)		135 (24.1)
Suburban	42 (37.5)	152 (33.9)		194 (34.6)
Rural	44 (39.3)	187 (41.7)		231 (41.3)
Total	112 (100.0)	448 (100.0)		560 (100.0)

Abbreviation: **RT**, radiotherapy; **CAD**, coronary artery disease.

**Note 1**: All above factors were used for propensity-score match to balance study groups.

**Note 2**: All *P* values were calculated by using chi-square test.

Fig. 1 shows flow chart of patient allocation. First, lung cancer patients were identified by using International Classification of Diseases, Ninth Revision, Clinical Modification (ICD-9-CM) code 162 (*n* = 32,613). Next, lung cancer diagnosis was validated by using a peer-confirmed data subset, i.e. Registry Data for Catastrophe Illness [13]. Then, we excluded patients with lung cancer history to identify newly diagnosed lung cancer patients (*n* = 21,436). Furthermore, we excluded 20,876 patients by using following criteria: distant metastases at the time of diagnosis (*n* = 10,871; ICD-9-CM codes, 196–199), chemotherapy used (*n* = 7,580), unpaired cases (*n* = 1,630), previous stroke (*n* = 780), and data error (*n* = 15).

Finally, we recruited 112 early stage lung cancer patients treated with surgery and postoperative radiotherapy into the surgery-plus-RT group. With a match ratio of 1∶4, propensity score paired 448 patients treated with surgery alone into the surgical-alone group. All data were validated by two independent database-specific biostatisticians, i.e. Miss Tsai and Hsu.

### Patients and Treatments

In clinical practice, postoperative chemotherapy was used in patients with positive mediastinum lymph nodes, i.e. pN1-3/stage II-III disease [17], [18]. Moreover, chemotherapy itself has been reported to enhance a risk of thrombosis-related vascular events in lung cancer patients [19]. Thus, the present study excluded patients treated with a component of chemotherapy. As a result, all included patients were early stage lung cancer patients treated with surgery plus radiotherapy or surgery alone. According to requirement of the Taiwan Health Insurance, major thoracic surgeries and radiotherapy were conducted by board-certified thoracic surgeons and radiation oncologists, respectively. In radiotherapy, postoperative irradiation alone was given in patients with positive surgical margin. For these patients, bronchial stump and high risk nodal stations of the mediastinum were irradiating targets [6], [17], [20]. In conventional fractionation, the highest dose was ranged from 45 Gy to 64.8 Gy according to individual department guidelines. These department guidelines were regularly audited by Taiwan Cancer Center Accreditation [21].

### Propensity score match: create comparable groups before analysis

In observation studies, it is crucial to reduce baseline imbalance before analysis. In this regard, we used propensity score match to pair cases [22]. Eight factors were match-paired before analysis: age [23], gender [23], hypertension [1], diabetes mellitus [24], coronary artery disease [25], atrial fibrillation [26], geographic region [27], and urbanization level [28]. With a ratio of 1∶4, a total of 448 surgery-alone patients were match-paired with respect to 112 surgery-plus-RT patients (Fig. 1 and Table 1).

### The primary endpoint and measurements

The present study defined two-year ischemic-stroke-free survival rate as the primary end point (ICD-9-CM codes, 433-438) [13]. All-type stroke-free survival rate was also calculated (ICD-9-CM codes, 430-438). Effective sizes of stroke occurrence were estimated by using univariate and multivariate analyses.

Urbanization level has been reported to affect incidence of stroke occurrence [14]. Thus, it was defined as one of independent variables. The following factors were used to determine urbanization level: population density, percentage of residents with a college or higher education, percentage of residents >65 years of age, percentage of residents who were agriculture workers, and number of physicians per 100,000 people. Radiation dose was estimated by using the charge code of external beam radiotherapy (code number: 36012B).

### Statistical analysis

Analysis were conducted according to the CONSORT statement [29], and STROBE guideline [30]. Two statistical packages, i.e. SAS (version 9.2; SAS Institute, Inc., Cary, NC) and SPSS (version 12, SPSS Inc., Chicago, IL), were applied, accordingly. Following statistical methods were used: Kaplan-Meier analysis to estimate stroke-free survival; log-rank test to assess curve difference; chi-square test to evaluate differences between category variables; and, Cox proportional hazard regression to conduct univariate and multivariate analyses for time-to-event endpoint. Independent factors that identified by multivariate analysis were used for stratified sensitivity analysis. To estimate an effective size, hazard ratio (HR) was provided with a 95% confidence interval (95% CI) in addition to a conventional *P* value. All tests were two-tailed and considered to be statistically significant when *P*<0.05.

## Results

### Study groups and patients

With a match ratio of 1∶4, a total of 560 patients were recruited into the following two study groups: surgery plus RT (*n* = 112) and surgery alone (*n* = 448; Fig. 1). All living patients were followed up for at least 2 years (median, 42.8 months; range, 24.8–72.8). Most patients were aged less than 65 years (*n* = 385, 68.8%) and male gender (*n* = 426; 76.1%). Propensity-score match balances eight baseline factors before analysis (Table 1).

### The primary endpoint: to explore a risk level of ischemic stroke occurrence

**Figure 2:**
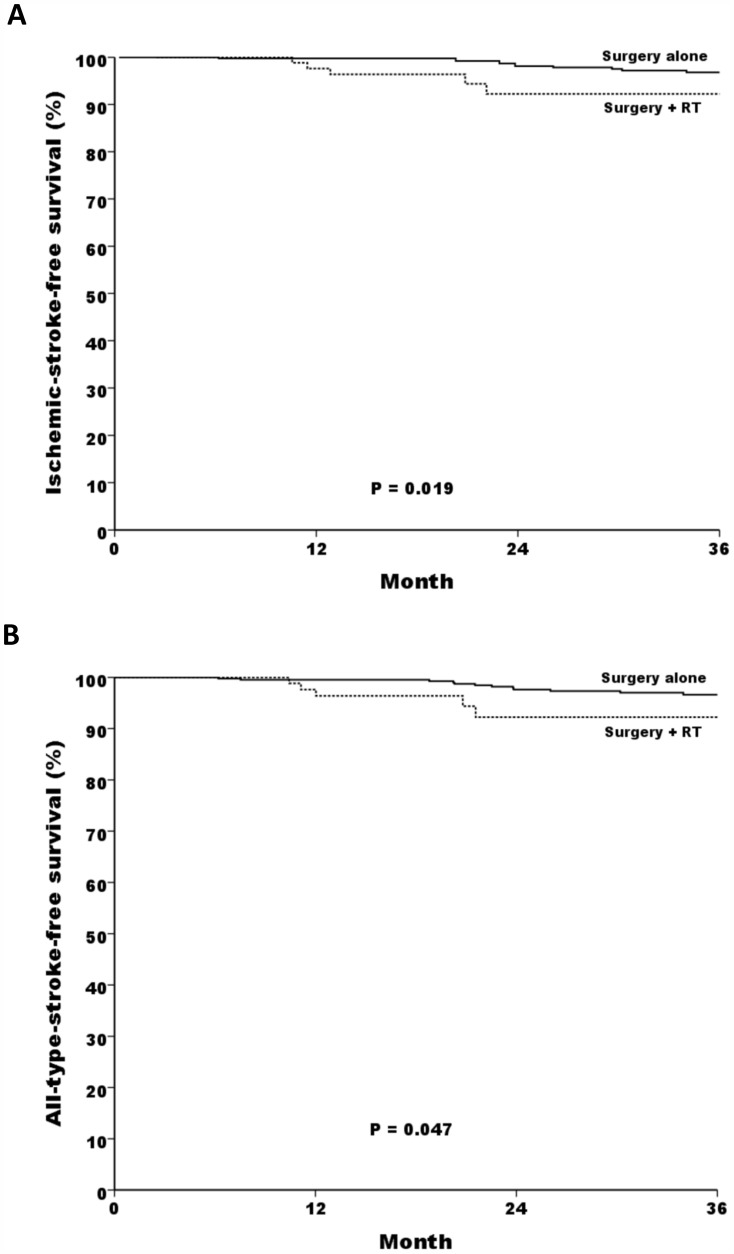
Kaplan-Meier estimates of 2-year stroke-free rates according to treatment groups (surgery-plus-RT versus surgery-alone). **Panel 2A,** ischemic-stroke-free rates: 92.2% versus 98.1%, *P* = 0.019; and **Panel 2B,** all-stroke-free rates, 92.2% versus 97.6%, *P* = 0.047.

Two observations supported a high incidence of ischemic stroke occurrence in surgery-plus-RT patients when compared with surgical-alone patients: first, a high ischemic-stroke incidence per 1,000 person-year at 2 years (22.3 versus 11.2, 1.99 folds) and at 5 years (24.4 versus 9.3, 2.62 folds; Fig. 1); and second, low 2-year ischemic-stroke-free and all-stroke-free survival rates, 92.2% versus 98.1% (*P* = 0.019; Fig. 2A) and 92.2% versus 97.6% (*P* = 0.047; Fig. 2B), respectively. In patients received radiotherapy, a higher estimated dose level seemly associated with a lower 2-year ischemic-stroke-free survival; however, log-rank test didn't support a statistical significance (89.8% versus 96.3%, *P* = 0.476).

### Multivariate analysis confirmed two independent risk factors

To explore risk level of ischemic stroke occurrence, univariate and multivariate analysis were conducted (Table 2). In univariate analysis, three factors showed a statistical significance (*P*<0.05): diabetes mellitus (*P*<0.0001), age (>65 vs. ≤65 years; *P* = 0.008), and treatment (surgery plus RT vs. surgery alone; *P* = 0.026). One factor demonstrated a statistical trend (0.05<*P*<0.10): chronic kidney disease (yes vs. no; *P* = 0.081). These four factors were used in further multivariate analysis.

**Table 2:** Hazard ratios for ischemic stroke occurrence according to predictive factors.

	HR (95% CI)	
	Univariate	Multivariate
Age (>65 vs. ≤65 years)	3.52 (1.38–8.94), *P* = 0.008*	2.40 (0.89–6.47), *P* = 0.078
Gender (male vs. female)	2.94 (0.67–12.80), *P* = 0.150	NA
Hypertension (Yes vs. No)	5.37 (0.71–40.62), *P* = 0.103	NA
Diabetes mellitus (Yes vs. No)	5.89 (2.33–14.85), *P*<0.0001**	5.02 (1.86–13.53), *P* = 0.001**
CAD (Yes vs. No)	1.63 (0.37–7.12), *P* = 0.511	NA
Atrial fibrillation (Yes vs. No)	1.86 (0.45–7.65), *P* = 0.387	NA
Geographic region (4 categories)	1.16 (0.43–3.12), *P* = 0.767	NA
Urbanization level (3 categories)	1.60 (0.51–5.05) *P* = 0.417	NA
COPD (Yes vs. No)	1.07 (0.19–5.87) *P* = 0.933	NA
Tuberculosis (Yes vs. No)	0.85 (0.28–2.59), *P* = 0.781	NA
CHF (Yes vs. No)	2.38 (0.76–7.39) *P* = 0.133	NA
CKD (Yes vs. No)	1.35 (0.87–2.09), *P* = 0.081	1.48 (0.17–10.27), *P* = 0.58
Post-OP RT (Yes vs. No)	3.28 (1.15–9.37), *P* = 0.026*	4.19 (1.44–12.22), *P* = 0.009**

Abbreviations: **HR**, hazard ratio; **95% CI**, 95% confidence interval; **vs**., versus;

**CAD**, coronary artery disease; **COPD**, chronic obstructive pulmonary disease;

**CHF**, congestive heart failure; **CKD**, chronic kidney disease; OP, surgery;

**RT**, radiotherapy; *, *P*<0.05; **, *P*<0.01.

**Note**: All *P* values were calculated by using Cox proportional hazard analysis.

**Figure 3:**
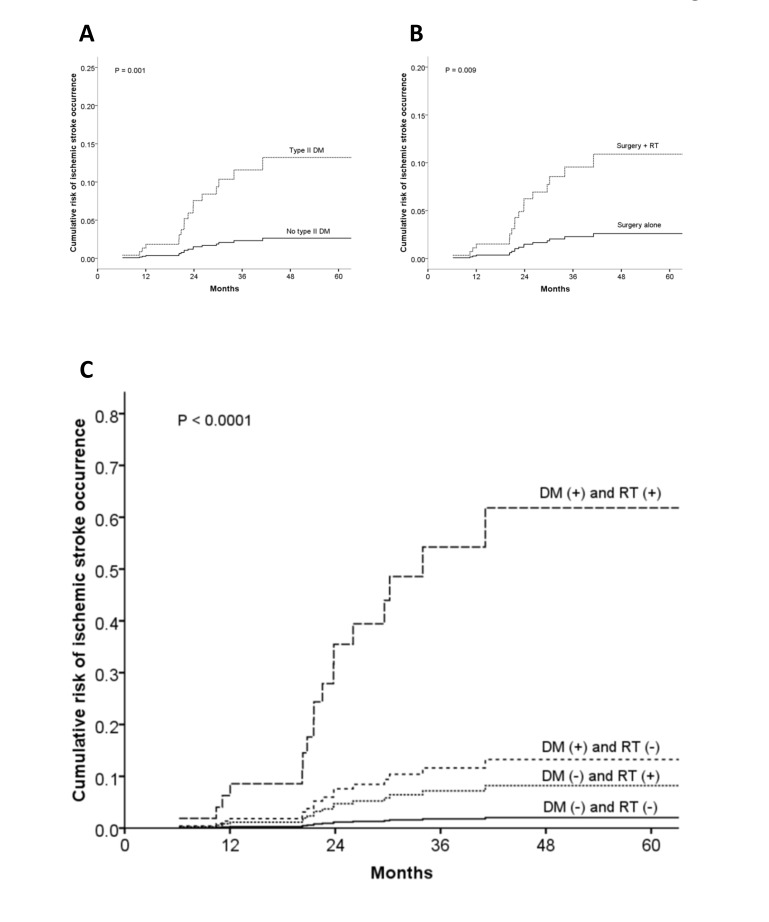
Cumulative risk estimates of ischemic stroke occurrence according to diabetes and irradiation. **Panel 3A**, adjusted HR in patients with type II diabetic mellitus (DM), 5.02 (95% CI 1.86–13.53; *P* = 0.001), when compared with those without type II DM; **Panel 3B**, adjusted HR in the surgery + RT group, 4.19 (95% CI 1.44–12.22; *P* = 0.009), when compared with the surgery alone group; and **Panel 3C**, the highest HR in patients with both DM and RT, 34.74 (95% CI 6.35–>100; *P*<0.0001), when compared with patients with both of none (reference value = 1).

Multivariate analysis identified two independent risk factors: diabetes mellitus (adjusted HR, 5.02; 95% CI, 1.86–13.53; *P* = 0.001); and, postoperative irradiation (adjusted HR, 4.19; 95% CI, 1.44–12.22; *P* = 0.009; Table 2 and Fig. 3A–B).

### Sensitivity analysis according to status of irradiation and diabetes

To further demarcate risk levels of ischemic stroke occurrence, we performed a stratified sensitivity analysis according to two independent risk factors: diabetes mellitus and postoperative irradiation. Table 3 shows a trend of increasing risk of ischemic stroke occurrence in the following four subgroups: non-diabetic and non-irradiated (HR, 1; as the reference); diabetic but non-irradiated (HR, 6.57; 95% CI, 2.21–19.54; *P* = 0.001); non-diabetic but irradiated (HR, 3.95; 95% CI, 1.97–7.92; *P* = 0.005); and most remarkably, both diabetic and irradiated (HR, 34.74; 95% CI, 6.35->100; *P*<0.0001). This observation may suggest an enhanced damage effect of irradiation on diabetic injured blood vessels (Fig. 3C).

**Table 3:** Hazard ratios for stroke occurrence in lung cancer patients stratified by treatment groups and diabetes mellitus.

	HR (95% CI)			
	The surgery-plus-RT group (*n* = 112)		The surgery-alone group (*n* = 448)	
	Diabetes	No Diabetes	Diabetes	No Diabetes
All-type stroke	27.33 (5.25->100), *P*<0.0001	4.05 (1.38–11.89), *P* = 0.010	4.90 (1.78–13.52), *P* = 0.002	1
Ischemic stroke	34.74 (6.35->100), *P*<0.0001	3.95 (1.97–7.92), *P* = 0.005	6.57 (2.21–19.54), *P* = 0.001	1

Abbreviations: **HR**, hazard ratio; **95% CI**, 95% confidence interval; **RT**, radiotherapy.

**Note 1**: All adjusted hazard ratios were estimated by using Cox proportional hazard analysis.

**Note 2**: Patients in the surgery-alone group without diabetes were as the reference (value = 1).

## Discussion

### Study question and main finding: a high risk of ischemic stroke occurrence was observed in irradiated lung cancer patients

In the literature, a high risk of ischemic stroke has been reported in irradiated cancer patients [2]–[4]. However, available data are limited in irradiated lung cancer patients. Recently, a high risk of ischemic stroke has been reported in lung cancer patients when compared with non-cancer controls: incidence per 1,000 person-year, 21.8 versus 15.1; and HR, 1.43 (95% CI, 1.34–1.51). Remarkably, this risk effect can be observed at a follow-up of two years [13]. These lines of evidence inspired the present study to explore whether a risk of ischemic stroke occurrence is also high in irradiated lung cancer patients.

In the present study, three observations supported a high risk of ischemic stroke occurrence in irradiated lung cancer patients when compared with non-irradiated patients: first, a high incidence per 1,000 person-year (22.3 versus 11.2, 1.99 folds); second, a low ischemic-stroke-free survival rate (92.2% versus 98.1%, *P* = 0.019); and third, a high adjusted hazard ratio (HR, 4.19; 95% CI, 1.44–12.22; *P* = 0.009). More notably, the highest risk of ischemic stroke occurrence was observed in diabetic-and-then-irradiated lung cancer patients (HR, 34.74; 95% CI, 6.35->100; *P*<0.0001).

### Biological reasoning: irradiation may further damage diabetic injured blood vessels

The present study generates a biological hypothesis: irradiation may further damage diabetic injured blood vessels and then increase a risk of embolic events in blood-supplied end organs.

Two lines of evidence supported this hypothesis. First, it is well known that diabetic vascular damages enhance a risk of thromboembolic events [24]. Second, clinical data suggested a rare but significant association of increasing ischemic stroke occurrence in irradiated cancer patients, such as head and neck cancers [2], breast cancer [3], and Hodgkin's lymphoma [4]. In these patients, regional nodal stations were irradiated, including the neck and/or mediastinum [5], [6]. In such a condition, ionizing radiation cannot be avoided to deliver on brain-supplied blood vessels [6], [7]. As a result, aberrant mural thrombosis may be generated, and a risk of brain stroke reasonably increased [31], [32].

The mediastinum was irradiated in patients with lung cancer and Hodgkin's lymphoma [6]; however, clinical data are largely lacking in irradiated lung cancer patients. Herein, the present study provided a population-based observation that a high risk of ischemic stroke occurrence may be associated with irradiated lung cancer patients, at least in a surgical cohort. Some molecular factors may play a role for this association, such as activation of reactive oxygen species [33]–[35], and nuclear factor kappa-B [35], [36]. However, detail biological interactions are largely unknown. Further studies are encouraged to investigate underpinning molecular mechanisms.

### A population-based surgical cohort is suitable to explore a risk level of ischemic stroke occurrence in irradiated lung cancer patients

To explore stroke risk in irradiated lung cancer patients, it is suitable to select a surgical cohort as study population [17], [37], [38]. Several reasons supported this viewpoint. First, lung cancer patients treated surgically demonstrated a technically resectable and medically operable status. This cohort feature avoided confounding effects from poor performance status and massive medical comorbidities. Second, lung cancer itself may increase a risk of stroke occurrence [13], [39], [40]. Thus, for comparing stroke risk in lung cancer patients, intra-cohort controls are better than non-cancer controls. In this regard, patients treated with surgical alone can be recruited as suitable controls in a surgical cohort. More notably, third, resected lung cancer patients demonstrated a longer survival than that of unresectable patients – this pattern allows an observation of stroke occurrence more likely.

### Study strength

Undoubtedly, randomized controlled trials are the gold standard in conducting clinical research [41]. However, population-based observation studies are useful in several conditions: first, to delineate what is achieved in the real medical world [42]–[44]; second, to explore an association of rare events [30], [45], [46]; and third, to approach issues that are difficult or infeasible to be investigated by using randomized controlled trials [43], [44]. In this regard, the present study applied a population-based design to investigate whether a risk of ischemic stroke occurrence is really high in irradiated lung cancer patients.

In clinical studies, regression model is useful to adjust confounding effects among multiple covariates [22], [47]; however, residual confounding may occur. Therefore, it is crucial to conduct an adequate case match before analyses. In this regard, propensity score match is recommended [43], [47]. After match, intergroup balance, study quality, and inference cleanness were improved in the present study. Moreover, sensitivity analysis is useful in investigating unmeasured confounding and hazard effects [15], [48]. The present study used a stratified sensitivity analysis according to independent risk factors. The highest risk of stroke occurrence was observed in diabetic-and-then-irradiated lung cancer patients. Further studies to investigate underlying biological mechanisms should be considered.

### Study limitations

The present study has several limitations. First, all included patients are anonymous in our database. Researchers cannot contact with individual patients or institutions to collect additional information, such as smoking habits, cancer stage, radiotherapy details, and body mass index. Thus, for overcoming this problem, we used COPD as a surrogate variable to represent, in part, smoking habits [15], [49]. Moreover, we excluded patients treated with chemotherapy. This exclusion omitted patients with positive mediastinum lymph nodes, i.e. those patients with pN1-3/stage II-III disease. Furthermore, we used radiotherapy charge code to estimate a radiation dose level.

Second, diagnosis is depended on ICD-9-CM codes in our database. Thus, the Taiwan Insurance Bureau regularly conducted external audits to maintain coding accuracy. Moreover, the present study used Registry Data for Catastrophe Illness for validation. Third, due to a limitation of case number, the present study cannot conduct full multilevel sensitivity analysis as that of a previously excellent study [15]. Instead, we performed a stratified sensitivity analysis according to independent factors. Fourth, patients taken with self-paid targeted therapies, such as Gefitinib (Iressa), cannot be identified. Although such cases were few during the study period, this may slightly affect our results.

Taken together, further prospective studies should be conducted to confirm our results. Nevertheless, the present study re-sheds us a light that population-based studies are useful in exploring a rare-event association and then to generate a biological hypothesis for further investigation.

## Conclusion

In a population-based surgical cohort, a high risk of ischemic stroke occurrence was observed in irradiated lung cancer patients, especially in those patients with diabetes mellitus. For these patients, close clinical surveillance and strict diabetes control should be considered. Further studies to define detail biological mechanisms are encouraged.
